# Understanding the cultural meanings of stroke in the Ghanaian setting: A qualitative study exploring the perspectives of local community residents

**DOI:** 10.12688/wellcomeopenres.14674.2

**Published:** 2018-11-14

**Authors:** Olutobi Sanuade

**Affiliations:** 1 Institute of Advanced Studies, University College London, London, WC1E6BT, UK

**Keywords:** stroke, social meanings, cultural meanings, community, Ghana

## Abstract

**Background:** Stroke has undergone different medical constructions over the years. While the medical profession posits that disease is a biological condition, universal and unchanging, social constructionists perceive illness as the social meaning of the biological condition. Even though the medical notion of stroke is monolithic and sometimes contradicts the representations by local community residents, little attention has been paid to understanding the cultural meanings of stroke. This study explores the cultural meanings of stroke in five different cultural settings across Ghana.

**Methods:** 30 focus group discussions (FGDs) were conducted with local community members in five communities (Ga Mashie, Tafo, Gyegyeano, Chanshegu and Agorve) located in five regions in Ghana. The FGDs were conducted in Ga, Twi, Fante, Ewe and Dagbani, and were transcribed verbatim into English. The transcripts were analysed thematically.

**Results:** The local words used for stroke in all the five cultural settings focused on physical disability associated with stroke after its onset, and this formed the dominant source of fear about the condition. Participants mentioned that spiritual and left-side stroke have the most debilitating impact on the sufferer. Although there was a general consensus that anyone can be at risk of stroke, there was a gender dynamics in the explanation of risk relativity. Participants believed that stroke can be cured through early detection and treatment, use of herbal medicines, and availability of financial resources. Compared to other disabling conditions, the community residents perceived stroke to be more severe due to the multifaceted disabilities associated with the condition.

**Conclusions: **This study showed that the social meanings of stroke in the five communities are multifaceted, and reflected co-existence of biomedical and cultural frameworks.  The findings showed the need to pay good attention to the sociocultural context when developing interventions strategies on stroke prevention and control in Ghana.

## Introduction

Over the years, stroke has undergone different medical constructions globally, and these influence perceptions and practice on management and prognosis. From the time of Hippocrates to the first half of the twentieth century, medical science mostly referred to stroke as “apoplexy”, meaning any sudden condition that began with loss of consciousness especially one in which the sufferer died within a matter of seconds after losing consciousness ( Cooke, 1824, cited in Pound *et al*., 1997). Hence, the global medical discourse was that apoplexy was a sudden acute event, with no hope of survival. For instance, Osler (1909), in his book on “principles and practice of medicine” made a suggestion that when an apoplectic sufferer is completely paralysed, the friends should be told at the onset that there is a slim chance of recovery, and in cases where the hemiplegia persists for more than three months and muscle contractures had developed, ‘it is the duty of the physician to explain to the patient, or to his friends, that the condition is past relief, that medicines and electricity will do no good, and that there is no possible hope of cure’ ( Pound *et al*., 1997). Even though there was pessimism about the prognosis of apoplexy, most especially during the 18 ^th^ century, physicians hoped for a future in medical advancement with prospects of better outcomes.

Towards the end of the 18 ^th^ century, there was a shift in the conceptualisation of apoplexy, and attention was paid to the characteristics that people with the illness shared, rather than focus on individual characteristics. In addition, even though there had been a distinction between apoplexies caused by obstruction and the ones caused by haemorrhage prior to this period, there was a new explanation that both conditions were caused by degeneration of the arterial wall ( Price, 1966). As a result, the term ‘cerebrovascular disease’ emerged in medical discussions and the use of the word ‘apoplexy’ faded away gradually ( Pound *et al*., 1997). Based on the medical construction of cerebrovascular disease, the emphasis on the person experiencing the condition began to fade away and ‘patients became less and less visible in medical texts’ ( Pound *et al*., 1997). The focus of the physicians shifted to the inside of the body of the sufferer, and the disease became more separate from the person in whose body it resided. During the 1950s, new treatment procedures such as angiography, cerebrovascular surgery and anticoagulants were developed for cerebrovascular disease and this formed the basis for a gleam of hope for better prognosis.

In spite of the fact that new treatment strategies for stroke were introduced during the 1950s, studies in North America showed that culturally informed ideas about aging shaped the provision of stroke care and the possibilities and perceived value of intervening. For instance, up to the 1970s, stroke was equated with ‘nothing more to do’ stage of a terminal illness and connoted hopelessness in a General Hospital in North America. According to Hoffman, ‘nothing can be done’ paralleled the concept of ‘nothing more to do’ when it came to stroke management and this was due to low education and training on stroke management as well as institutional policies and professional values that minimized the benefits that health professionals could obtain through the care of stroke patients ( Hoffman, 1974). This, however, influenced staff attitudes towards stroke patients and they either withdrew from participation in care of these patients or denied the complications of stroke by managing patients in a way that concealed from them the chronicity of their disability. They let the patients believed that they could recover completely if only they tried enough because the responsibility for getting well lied with them (i.e. patients) and not physicians. Hoffman explained that belief in the possibility of complete elimination of disability delayed patients from making ‘satisfactory long-term adjustment to their condition’ ( Hoffman, 1974). As a result, some of the rehabilitation strategies were unsuccessful because patients had the perception that they could get around their disability since rehabilitation could only enable them to live in spite of disability, and not without it. Nevertheless, research shows that stroke rehabilitation do not promote actual physiological recovery, but only enables patients to fully use whatever function remains post-stroke, to gain strength and endurance ( Kaufman & Becker, 1991).

Although the word 'stroke' firstly occurred in English literature in the late 16th century (1599) ( Pound *et al*., 1997), it became widely used from the 1950s. The term ‘stroke’ was used to capture the sudden nature of an acute event, and was adopted at the time when the initial surgical or drug treatment for the condition was dashed. In 1970, a new Journal called “STROKE” was developed to synthesise evidence on stroke in a way that would help physicians across the world get access to updated information on stroke ( Millikan & Clarke, 1970); this journal probably increased the use of the word “stroke”. Through medical advancement, stroke is now seen as a condition of vascular origin but with hope of rehabilitation through physiotherapy and occupational therapy ( Peszczynski *et al*., 1972; Walker *et al*., 2013). The World Health Organization therefore define stroke as “rapidly developing clinical signs of focal (or global) disturbance of cerebral function, with symptoms lasting 24 hours or longer or leading to death, with no apparent cause other than of vascular origin” ( Truelsen *et al*., 2000).

Even though the medical construction of an illness is important for management and prognosis, perceptions of local community residents are equally important, albeit often different from the medical perception. Sociologists believe that there are complex relationships between the medical and cultural notion of disease. Social constructionists particularly posit that individuals and groups produce their own conceptions of reality, and that knowledge itself is the product of social dynamics. For the medical profession, disease is a biological condition, universal and unchanging; however, social constructionists perceive illness as the social meaning of the ‘biological’ condition. That is, diseases are not just biological conditions, they have social meanings ( Sontag, 1978) and these exist for social rather than pure biological reasons ( Conrad & Barker, 2010).

The social meanings of illnesses shape how people make sense of and respond to an illness. Ervin Goffman argued that some illnesses are stigmatised and considered disabilities while others are not ( Goffman, 2009). Despite the fact that there is nothing inherent in an illness that makes it stigmatising, the social meanings acquired through social interactions makes a condition stigmatised or considered as disabilities unlike others ( Goffman, 2009). The cultural meanings of illness are therefore important because they have an impact on the way the illness is experienced, how the illness is depicted, the social response to the illness and what policies are created concerning the illness. The cultural meanings also shed some light on the rationale for noncompliance which is relevant for developing context-centered strategies to improve effective implementation of medical treatment ( Conrad & Barker, 2010).

While the medical notion of stroke has already been established, the cultural meanings of stroke have received little attention. Allan Young particularly argued that there is usually a difference between diagnosis of an illness as part of the biomedical model and illness experience as part of the sociological or anthropological understandings and experience of such illness ( Young, 1976). In British Colonial Africa, despite the fact that biomedical knowledge played an important role in the wider creation of knowledge of ‘the African’, many biomedical theories and interventions failed because no consideration was given to the social and political context ( Vaughan, 1991). A few existing studies in some parts of Africa showed that people draw on both medical and supernatural frameworks of understanding to explain stroke and its associated disruptions to individual biographies ( Legg & Penn, 2013; Mshana *et al*., 2008a; Mshana, 2008b; Penn, 2007; Penn *et al*., 1992; Sanuade, 2016), and communities regard both the clinical and social diagnostics as important in stroke management ( Hundt *et al*., 2004). The aim of this study was to explore the cultural meanings of stroke in five communities across Ghana. Specifically, this study explored perceptions on stroke definition, types, cure, ‘group at risk of’, and general community ideas. Findings from this study can help to understand how stroke is situated within the Ghanaian cultural context, and this is important for developing intervention strategies on prevention and management.

## Methods

### Design

**Figure 1.**
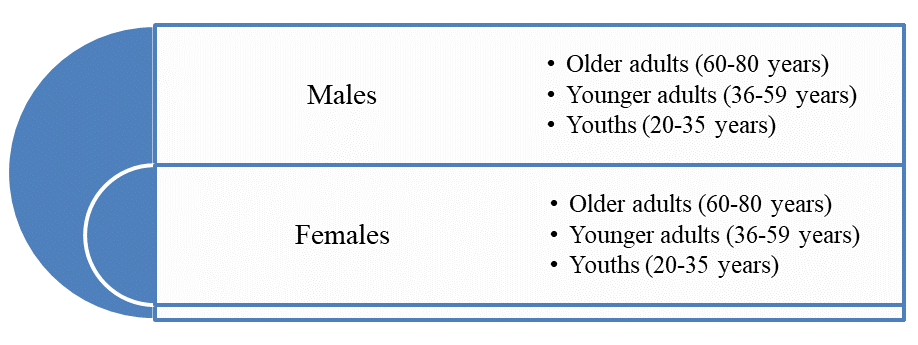
Segmentation of the focus group discussions by age and sex.

This was a cross-sectional qualitative study. Data were gathered through focus group discussions (FGDs) with local community residents and none of these participants had stroke at the time of the data collection. Six FGDs were conducted in each of the study sites and participants were segmented by age and sex ( Figure 1). A total of 30 FGDs were conducted in five communities across Ghana through a purposive sampling technique. Participants’ profiles are presented in Table 1. The number of participants in each of the group discussions ranged from six to ten. Research has shown that this range is adequate to facilitate informative conversation and for the group discussion not to become disorderly or fragmented ( Krueger, 1994; Nyumba *et al*., 2018). The Standards for Reporting Qualitative Research (SRQR) guidelines were used in this study ( O’Brien *et al*., 2014).

**Table 1.** Participants' demographics.

	Ga Mashie	Agorve	Gyegyeano	Tafo	Chanshegu	Total (n=255)
**Age**						
18–34	18	16	19	20	15	88
35–59	18	18	17	17	20	90
60 and above	16	16	12	17	16	77
**Sex**						
Male	24	26	23	25	25	123
Female	28	24	25	29	26	132
**Education**						
None	7	13	3	3	36	62
Primary	5	12	2	6	3	28
Secondary	38	24	37	42	12	153
Higher	1	1	4	3	0	9
No response	1	0	2	0	0	3
**Occupation**						
Unemployed	11	6	9	18	3	47
Trading	14	8	12	10	20	64
Farmer	4	21	0	1	24	50
Artisan	10	3	9	11	1	34
Others	13	8	5	14	3	43
Retired	0	4	10	0	0	14
No response	0	0	3	0	0	3
**Marital Status**						
Never married	29	14	22	17	8	90
Married	14	33	18	16	43	124
Formerly married	9	3	8	12	0	32
No response	0	3	4	2	0	9
**Religion**						
Christian	44	30	41	50	1	166
Muslim	3	0	7	3	50	63
Traditional/ others	2	9	0	0	0	11
None	3	1	0	0	0	4
No response	0	5	0	6	0	11

### The study context

Ghana is a multi-ethnic, multi-religious and multi-cultural country with ten administrative regions. Its current population, which is estimated at about 25 million ( Ghana Statistical Service, 2012), is a mix of large and small ethnic groups. Some of the major groups in the country include the Asante, Fante, Ga, Ewe and Dagomba. Even though people from various ethnic groups are dispersed across the country due to easy geographical and social mobility, many of these people still retain their cultural identities ( Asante & Gyimah-Boadi, 2004).

The Burden of Disease Study conducted in 2016showed that stroke is the eighth leading cause of death in Ghana and a major contributor to the Disability Adjusted Life Years Lost (DALYs) ( Ghana Ministry of Health, 2016). Evidence also suggests increase in stroke prevalence and incidence in the country. Despite the increase in stroke morbidity and mortality ( Agyemang *et al.,* 2012), there is limited evidence-based stroke services and low priority for stroke care in Ghana. The health system landscape in the country is such that healthcare delivery is provided by the formal medical healthcare system as well as informal non-medical healthcare such as faith-based and ethno-medical systems. Out of these healthcare delivery systems, the formal medical healthcare system is seen as the only ‘legitimate’ healthcare system set up for stroke management and control. Even though there are tertiary teaching hospitals, regional hospitals, district hospitals and sub-district health centres in the country, the district hospitals and sub-district health centres often have limited clinical capacity for stroke care. Also, the referral hospitals which are supposed to be well equipped for stroke management and control often lack adequate capacity for acute stroke care. For instance, there is only one stroke unit in the country. Out of the 11 major referral hospitals in the country, 7 hospitals have functional computed tomographic (CT) scan and only one hospital have functional magnetic resonance imaging (MRI) scan services ( Baatiema *et al.,* 2017). In addition, none of these hospitals has an occupational or a speech therapist to assist in providing acute stroke care ( Baatiema *et al.,* 2017).

### Study communities

**Figure 2.**
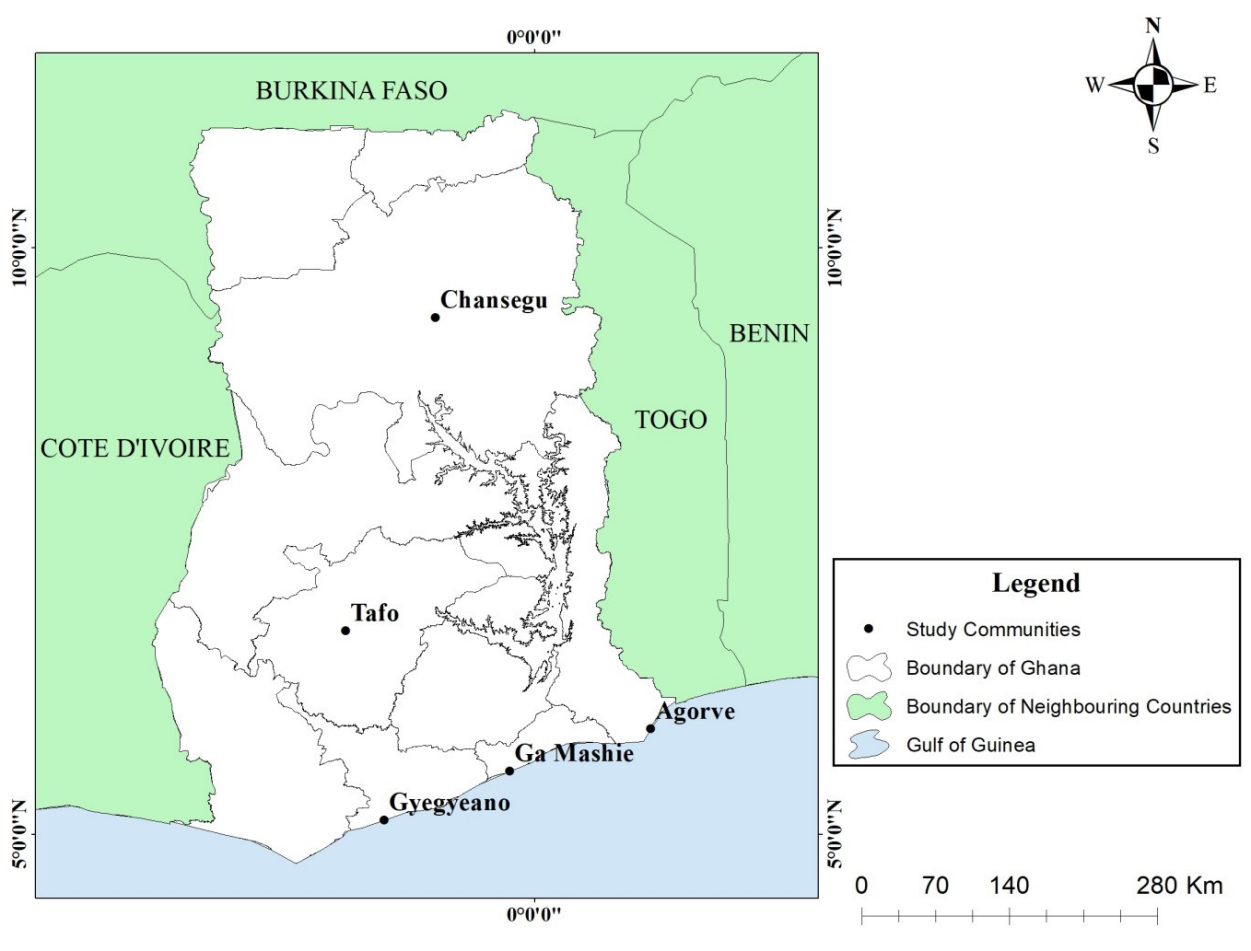
Map of Ghana showing the five study communities.

Data were collected in five communities (Ga Mashie, Tafo, Gyegyeano, Chanshegu and Agorve) located in five regions in Ghana (Greater Accra, Ashanti, Central, Northern and Volta regions, respectively) ( Figure 2). Participants were selected from these communities in order to capture nuances in stroke perceptions from the major ethnic groups in Ghana.

Ga Mashie is located on the Atlantic Coast of the Greater Accra Region of Ghana. It is home to the Ga people who speak the Ga language. The area is referred to as ‘Old Accra’ because it is the place where the original Ga ethnic group first settled; this makes Ga Mashie the oldest community in Accra ( Mahama *et al*., 2011). Ga Mashie, constituting James Town and Ussher Town, covers an area of 100 hectares along the southwest coast of Accra. The population of Ga Mashie has grown rapidly over the years and it is currently one of the most densely populated communities in Accra ( GSS, 2012). It is characterised by low income, low levels of education, inadequate access to improved sanitary facilities, low access to healthcare services, high population density and double burden of communicable and non-communicable diseases (NCDs) ( Awuah *et al*., 2014; Mahama *et al*., 2011). Furthermore, there have been several NCD studies and interventions in these communities ( Awuah *et al*., 2014; de-Graft Aikins *et al*., 2014; Sanuade, 2016) and this indicates that the residents may have more NCD knowledge compared to the other communities. Additionally, there was previous contact with Ga Mashie residents and this made recruitment easier compared to the rest of the communities.

Tafo is a town in Kumasi Metropolitan District in the Ashanti Region of Ghana. The largest ethnic group in this city is the Asante, a sub-group of the larger Akan ethnic group, and the population speaks the Twi language. Although there is ethnic and cultural diversity in this community, the population is close-knit due to the presence of a strong traditional administrative set-up that fosters cohesion among the diverse ethnic groups. The community is densely populated, has low level of education, and low socio-economic status, and poor sanitation

Gyegyeano is located in Cape Coast in the Central region of Ghana. It was founded by the people of Oguaa. It is one of the most historic cities in Ghana and has had a lot of interactions with the Europeans (the British, the Portuguese, the Swedish, the Danish and the Dutch). It is home to the Fante people, a sub-group of the larger Akan ethnic group who speak Fante. The city has some of the Ghana’s best secondary and Technical schools and so attracts people from across the country.

Chanshegu is located in Tamale, which is one of the six Metropolitan Assemblies in Ghana and the only Metropolis in the three Northern regions (i.e. Upper East, Upper West and Northern regions). Chanshegu is occupied by an ethnic group called the Dagombas; they are the largest ethnic groups in Tamale and speak Dagbani. This community has a traditional administrative head who is the leader of the community. Although the community is sparsely populated, the people are close-knit and are predominantly Muslims. It is characterised by low income, low level of education, poor sanitation, and low access to healthcare services.

Agorve is located in Keta, a town in the Volta Region of Ghana. It is the capital of the Keta Municipal District. The area was an important trading post between the 14th and the late 20th centuries. It was settled by the Anlo Ewe, a sub-group of the Ewe people who, in the 17th century, migrated to the area from Ketu, in Benin and speak ‘Anlogbe’, a dialect of the Ewe language. They are a patrilineal society governed by a hierarchical, centralized authority.

### Data collection

Data collection took place between October and November 2017. Data collection tools were developed to examine the cultural meanings of stroke among five dominant ethnic groups in Ghana (Ga, Ashanti, Fante, Ewe and Dagomba). The sociodemographic characteristics of participants were collected using datasheets ( Supplementary File 1). The FGDs were conducted in local languages (i.e. Ga, Twi, Fante, Ewe and Dagbani) by Thirteen (13) trained interviewers [Ga Mashie (n=2), Tafo (n=3), Gyegyeano (n=2), Chanshegu (n=3), and Agorve (n=3)]. The group discussions lasted between 90 and 150 minutes and were guided by the FGD guide ( Supplementary File 2). Sample of the questions that were asked are provided in Box 1. Ethical approval was obtained from the University College London Research Ethics Committee (REC) with approval number 11371/001, and locally from Ghana Health Service Ethics Review Committee (GHS-ERC Number- 007/09/17). The aims of the study were explained to the participants and written consent was obtained from all of them. Permission to record the group discussions was obtained from the study participants prior to the start of the group discussions. Confidentiality was strictly observed. At the end of the data collection, the recordings were transferred from audio recorders to a computer and were deleted after the extraction. Currently, they are being stored on a secure laptop and will be deleted on October 16 2018.

Box 1. **Sample questions from the focus group discussion guide**

1. How will you define/describe stroke?

a. What is/are the ‘local’ word(s) used to describe stroke in your dialect and what do these mean?

2. Do you think stroke can be cured? *(Explore reasons)*

3. Which group of people are most likely to get stroke? *(Explore reasons)*

    
***Prompts**: aged, men, women, young and old, etc.*

4. What kinds of ideas or beliefs do people in this community have about stroke?

5. What do you fear most about stroke?

6. Do you think there is a difference between people living with stroke with different disabilities (physical, psychological, cognitive, behavioural) and people living with other forms of disabilities (physical, psychological, cognitive, behavioural) but are not living with stroke? *(Explore reasons)*

### Data analysis

The analysis was performed after all the group discussions were conducted, transcribed and quality checked. All interviews were transcribed verbatim from Ga, Twi, Fante, Ewe and Dagbani into English language by a team of transcribers with Ga, Twi, Fante, Ewe, Dagbani and English language competence. Data were checked and cleaned, stored as Microsoft Word files (using MS-Word, 2010) and exported to ATLAS TI7. Thereafter, data were analysed using thematic approach ( Attride-Stirling, 2001). The first stage of analysis involved reading all the transcripts to identify emerging codes and themes. A mix of deductive and inductive codes was employed for the analytical framework. The deductive codes were derived from previous studies on stroke reviews ( Karenberg, 1994; Morris, 2011; Pound *et al*., 1997), and conceptual ideas were drawn from the social construction of illness theory ( Conrad & Barker, 2010). Several discussions were held with a colleague, an expert in African history, so as to develop an elaborate codebook that captures social meanings of illness in an African setting. The second stage of the analysis involved identifying the linkages between codes, themes, and appropriate respondent quotes, and existing research. The interpretive process was shaped by the social construction of illness framework to highlight the cultural meanings and description of stroke by the five communities.

## Results

The findings are grouped under seven themes: 1) local words for stroke and their meanings; 2) community ideas on stroke; 3) typology of stroke; 4) perceptions on cure of stroke; 5) groups at risk of stroke ; 6) most fearful thing(s) about stroke, and; 7) stroke and other disabling conditions.

### Local words for stroke and their meanings

Participants were asked to provide the local words used for stroke, and what these meant. Their responses are summarised in Table 2. The medical English word ‘stroke’ was used during the group discussions and participants were asked to provide the local words ( Box 1). Also, many of the community members were familiar with the word ‘stroke’ and could recognise who a stroke survivor is in line with medical descriptions. In the five communities, stroke was described as: 1) a debilitating or paralytic illness; 2) one leg one arm; 3) a ghost illness, and; 4) a shivering illness. All the participants referred to stroke as a ‘paralytic or debilitating illness’ because it is an illness which breaks the sufferer down by causing loss of body functions and incapacitation. Stroke was also described as *‘*one leg one arm’ among the Ga (in Ga Mashie) and Ewe (in Agorve) because after its onset, the sufferer either experiences weakness at the right or the left side (i.e. arm and leg). In addition, Ga Mashie participants referred to stroke as a ghost illness *“sisa hela”*; they mentioned that this term was developed in the 1960s when community knowledge on causes and management of stroke was low. *Sisa hela* (ghost illness) is generally used among Ga to refer to any illness that people don’t know how it came about and curing it is difficult. Finally, Ga Mashie participants referred to stroke as “ *kporkpormorhela”* which literally means epilepsy in Ga; this is because the onset of stroke triggers uncontrollable shaking similar to epilepsy.

**Table 2.** Local words for stroke and their meaning.

	Meaning	Ga	Twi	Fante	Ewe	Dagbani
1	Debilitating or paralytic illness	*Kumormorhela/* *kumorhela*	*mbubuo*	*Ndwe dwee/* *Mbubui*	*gbagbador*	*Gbali ni* *boug*
2	one leg one arm	*nane kome nine*	-	-	*akpadekador*	
3	Ghost illness	*sisa hela*	-	-	-	-
4	Shivering illness	*kporkpormorhela*	-	-	-	-

### Community ideas about stroke

Participants were asked to state the kinds of ideas or beliefs that people in their communities have about stroke. Four different ideas emerged from their narratives. In Ga Mashie, stroke was referred to as a ghost illness due to lack of knowledge on causes and management. It was also a belief in the community that stroke occur as a result of being cursed.

*…like we told you earlier, people say it is “ghost sickness” and people are cursed with it. That is what they say. You will not hear people say it is a normal illness like the way they say malaria is. Anytime someone gets it what they say is the person has being cursed by someone or it is the person’s bad way of living which has made him to be affected by it or the person’s ancestors have cursed him [Young women, Ga Mashie]*

In Gyegyeano, participants mentioned that community members refer to stroke as a spiritual illness caused by witchcraft and ghost activities. The community also had the belief that stroke occurs as a result of having sex while standing as well as engagement in strenuous work. The community idea in Agorve was that stroke causes early death, most especially if it occurs on the left side of the sufferer.

*Unfortunately for us in Africa, it’s only the older ones, the old women that are regarded as witches. People will say that old lady at that place is the cause of your illness. Then they will go and expel that woman from the house to another place. Meanwhile, she doesn’t know about you. So that is the belief. The moment there is a chronic illness or disease in the family, then they attribute it to someone who is causing it. That is the belief and then they consult a spiritualist. And then he too, because he wants money, he will endorse it [Elderly men, Gyegyeano]*

Those from Chanshegu mentioned several pluralistic ideas about stroke. First, there was the idea that stroke affects only those who are strong, and these are mostly men. They mentioned that some people in their community also have the idea that stroke can affect anyone irrespective of whether they practice good lifestyles or not. There was also the belief that stroke can only attack someone in the midst of other people. That is, stroke does not affect the sufferer when he/she is alone. In addition, some of the community members believed that stroke is not a hospital illness as it can only be managed with local herbs. Finally, stroke was seen as the handiwork of God in this community. That is, stroke occurrence is neither due to the weakness of the sufferer nor his or her lifestyles, but it is a divine work of God, and this is why the place for stroke management is not the hospital.

*As he has just said, many have told us that stroke does not like injection. For stroke, it is believed that its place is not the hospital but the local herbs. If you try the herbs and God permits, you will be successful [Adult men, Chanshegu]*
*But we don’t look at it like the person is useless that is why he was affected but we see it as a divine work from God. It’s not because of the weakness of the person that it affected him. And it is not because of his strength that it affected him [Young men, Chanshegu]*

### Typology of stroke

When participants were asked to describe stroke in their local contexts, typology of stroke emerged as a thematic area of discussion. This typology broadly focused on stroke causation and characteristics. With respect to causation, participants from Agorve and Gyegyeano differentiated between natural and spiritual stroke, although there were contradictions in their explanations. They mentioned that natural stroke is caused by hypertension while spiritual stroke is caused by witchcraft activities, sorcery and curses. There was the belief among those from Agorve that while natural stroke is difficult to manage, spiritual stroke can be easily cured. They particularly mentioned that:

*..the spiritual stroke is very easy to cure. There’s a young man in the area like that. He is a student at KNUST, through much prayers, he’s now free of stroke; but if the stroke comes on you naturally, then there is no way you can cure it and if you don’t have money, you will be dead in no time [Young male group, Agorve]*

On the contrary, those from Gyegyeano mentioned that while natural stroke is easy to manage, spiritual stroke is difficult to manage because no matter the amount of medicine taken, the stroke will eventually kill the sufferer. This was evident in the narratives of the adult male group.

*…there are some orthodox medicine as well as traditional medicine for natural stroke. It’s the spiritually caused stroke which is dangerous. No matter the kind of medicine you take, the stroke will still kill you [Adult male, Gyegyeano]*

Regarding stroke characteristics, participants from Ga Mashie and Gyegyeano explained the difference between mild and severe stroke. They mentioned that while mild stroke affects some parts of the body, severe stroke affects all parts of the body and makes the sufferer bedridden. One of the participants from the young women group in Gyegyeano said that:

*I know there’s something called mild stroke. A friend of mine had one. All of a sudden he couldn’t walk straight and then his mouth was shaken off balance too. His hands still had power. This is what I know to be mild stroke. I was told someone in his workplace caused it spiritually. I don’t know whether he’s ok now… Then there’s the severe one. With this one, the person is bedridden. He’s not able to walk or do anything [Young women, Gyegyeano].*

Further, a distinction was made between right-sided and left-sided stroke. Participants from Tafo and Agorve explained that the stroke which occurs on the right-side of the sufferer’s body can be cured while the one which affect the left side is more dangerous and can lead to early death.

*There are two types of stroke: one affects your right half (arm and leg) and the other affects the left half of your body. If the stroke affects the left side of your body then you will die within 8 months…But for some people, the stroke affects only the right side. They are able to talk. It’s easy to be cured if you have only your right side affected …. [Adult men, Tafo]*

However, one participant among the adult male group in Tafo disagreed with the rest of the group members and said that the severity of stroke is not about the side in which it affects, but the ability to seek proper management.

*Well I don’t agree with what he just said. It’s not really about the stroke affecting your left side or your right side. It all depends on how you will treat it. Sometimes the stroke affects only the brain and kills the person without affecting any half of the body. So for me, it’s not about whether it has affected the left part of your body or the right part of your body [Adult male group, Tafo]*

### Cure of stroke

Participants were asked to explain whether stroke can be cured or not. The dominant response in all the communities was that stroke can be cured. Particularly, they mentioned that stroke can be cured if: 1) there is money -because stroke is rich people’s illness; 2) there is early detection and treatment; 3) the sufferer manages the stroke with herbal medicines, seek treatment from traditional healers, or practice faith healing, and; 4) the sufferer adopts good lifestyle practices such as physical activity and good dietary patterns.

*Stroke can be cured with money. If you don’t have money you can’t cure it…it is rich people’s illness…if there’s no money, then it will break you down. [Adult women, Ga Mashie]*
*It depends on how early the person is able to detect the sickness. For example, I know a man who detected early that he has stroke. So he was taken to the hospital immediately. As we speak, he’s completely healed.So early detection makes it easy for you to be cured. If you don’t detect it early and you also don’t have anyone to take care of you while at home, it will become difficult for you to be healed. I hope you agree with me? [Young women, Gyegyeano]*
*On curing it, we have certain herbs we use to treat it. If we try it and God permits we will be able to cure it [Adult men. Chanshegu]*

Box 2. **Perceptions on cure of stroke**

***Interviewer***: *hmmm. Can stroke be cured?*

***Respondent***: *yes*

***Interviewer***: *do we all agree?*

***Respondent***: *yes, we all agree*

***Interviewer***: *in what way can stroke be cured?*

***Respondent***: *the person must be put on a special diet. He must walk around often and must not wear any foot wear. When your foot touches the ground it strengthens the bones in your legs and helps in healing. The person is given medicine and also put on a special diet with specific time of eating. If the person can abide by the diet it helps in the healing [Young women, Tafo]*

On the contrary, elderly women groups in Ga Mashie, Agorve and Chanshegu mentioned that stroke cannot be cured if it affects the left side of the sufferer, or if the sufferer falls on the ground during the stroke onset or seek treatment from the hospital. It was also believed among the young groups in Ga Mashie and Gyegyeano that stroke cannot be completely cured because the sufferer does not return to the normal state.

*Yes. Stroke can be cured after a long time but the stroke patient wouldn’t be able to walk the same way again or your hand could twist a little. Some also drag their feet. The way you used to speak changes [Young men, Gyegyeano]*
*You can be cured but something about you will change. It’s either in your walking or something else. It won’t be as it used to be. Your hand can be twisted [Young women, Gyegyeano]*

### Groups at risk of stroke

Participants were asked to mention the groups that are most likely to get stroke. There was a general consensus in all the group discussions that anyone can be at risk of stroke if such individual engages in poor lifestyles. They particularly mentioned that some decades ago in Ghana, it used to be only the elderly that were affected by stroke; however, everyone is at risk of stroke now due to change in dietary practices and food toxicity. For instance, some of the participants said that:

*These days, children even get stroke. Children get stroke these days. Several years ago elderly people who were in their sixties, seventies, and eighties were those who got stroke. But these days children get stroke [Adult men, Gyegyeano]*
*In the past, this illness (stroke) wasn’t common. But now even little children suffer from it. It is from the kind of food we eat; I’m not too sure…Maybe the chemicals which are now in our foods which eventually get into our bodies [Elderly men, Agorve]*

Although it was mentioned that anyone is at risk of stroke, participants were of the opinion that stroke cases are higher among the elderly. There was however the notion that even though the elderly are more affected by stroke, the prevalence is low among those who are 80 years and above. In addition, even among the elderly, participants mentioned that men are more at risk of stroke than women because of: frequent thinking/worrying due to marital breakdown and joblessness; indulgence in indiscriminate sex such as having multiple sexual partners and having sex while standing, and; the notion that stroke affect those who are strong and men are usually the strong ones.

*The people I usually see having this disease are men. And the reason why I see men getting this disease is that usually when their wives divorce them, they think often. When it so happens, they get mild stroke. Another reason is that a man may be working and earn some income, but when he is sacked, he will resort to thinking, then he will get mild stroke. Sometimes too, a man may be actively looking for a job and it worries him that he is unable to afford his rent for example…so he will be thinking about this and then get mild stroke [Adult men, Ga Mashie]*
*There are some men who are fond of women. They could sleep with about 10 women in a single day. They claim that is evidence that they are indeed men. So the men exert all their energy during sex. For the woman she does not exert a lot of energy like the men. After some time these men become paralyzed…[Adult men, Tafo]*
*Ok, for stroke, it is very prevalent among men. It mostly attacks those who are strong… It does not attack the weak [Adult women, Chanshegu]*

Further, the young women group in Gyegyeano mentioned that the reason why women are less likely to get stroke is because their menses act as a protective factor.

*I learnt that when women go through their menses, all the waste products in the blood is expelled and that’s why they aren’t likely to get stroke as men [young women, Agorve]*

On the contrary, the elderly women group in Cape Coast mentioned that stroke is common among women because women experience a lot of stress and worrying compared to men.

*Stroke is common among women because women worry a lot more than men.. in our daily activities, we carry too much heavy loads. When it gets to a point in time that practice brings sicknesses upon us [Elderly women, Agorve]*

### Most fearful things about stroke

The dominant theme on what participants fear most about stroke was the physical disability associated with the illness. All the groups mentioned that they are afraid of post-stroke physical disabilities such as mouth distortion, speech impairment and paralysis which confines the sufferer to one place and incapacitates such individual not to engage in any activity. The adult women group in Tafo mentioned that their fear of stroke is that it breeds poor interpersonal communication by making it difficult for the sufferer to communicate their needs to others.

Further, the sudden change in the biography of the sufferer constitutes the fear for some of the participants. Particularly, they mentioned that they are frightened when they see a stroke survivor and this is because the sufferer may be moving about normally at one moment and suddenly, the person has a stroke and the entire body functions change. One of the members of the adult women group in Gyegyeano said that:

*Yes, when I see a stroke patient I become frightened. This is because some few days ago the stroke patient was moving about normally now he’s paralyzed in some parts of the body. The sudden change really scares me [Adult women, Gyegyeano]*

In addition, participants said that they fear the psychological disability associated with stroke and this is because after the stroke onset, the sufferer engages in frequent worrying and thinking due to changes in the physical body and life trajectory. Inability to cater for family due to stroke complications featured in all the group discussions in Chanshegu. These participants mentioned that the onset of stroke makes it difficult for the sufferer to take care of his/her family because of restrictions in activities of daily living. As a result, there is a serious effect on the economic circumstances of the entire household and children’s education and general well-being are affected.

Some of the participants from Agorve mentioned that what they fear most about stroke is the fact that it brings about changes in dietary patterns/practices. They mentioned that a stroke survivor may no longer have the opportunity to indulge in some foods which he/she likes. For instance, the elderly male group said that:

*..when you get stroke, there are certain foods you can’t eat anymore. So that is what frightens us about it [Elderly men, Agorve]*

Furthermore, the young men group in Chanshegu said that one of the things they fear most about stroke is the fact that it can affect anyone. Finally, some of the participants mentioned that the fact that stroke eventually causes death makes them afraid. The belief for some people was that no matter how much an individual manages the condition, the person will eventually die as a result of the condition.

*My prayer at all times has always been against stroke. If I am sitting and it comes to mind, I always feel like crying. Ever since I grew to a certain age if even some is cured of stroke, by and by the person will not survive for long. No matter how cured the person will be it will get to a point that you will see that the person has died as a result of that same illness. So for this illness, we are just praying to God that wherever it’s cure will come from to help us. It will be good because if you hear that someone has been treated of stroke and is cured, by and by the same ailment will be back again [Adult men, Chanshegu]*

### Stroke and other disabling conditions

Participants were asked to explain if there is a difference between people living with stroke with post-stroke disabilities and people living with other forms of disabling conditions. Participants differentiated between post-stroke disabilities and other disabling conditions such as mental disability (e.g. psychosis) and physical disabilities (e.g. impaired-eye and other physical deformities). The first category of people believed that post-stroke disability is better than mental disability. For instance, with respect to stroke-induced disability and psychosis, participants mentioned that although the speech of the stroke sufferer may be impaired, he/she will still be able to hold meaningful conversation with others; however, a psychotic person finds it difficult to hold logical communication with others.

*Someone with psychosis will speak alright but it won’t make sense. You will hear everything he says but his speech will not make sense... as for stroke, the person’s speech makes sense. Since he can see, he can say that what you are doing is wrong but you might not hear him so you need to listen to him well. You might not hear him properly but you need to pay attention to what he says [Young men, Ga Mashie]*
*..the stroke patient can fetch water but for the mentally ill person, if you tell him to go and fetch water, it would be a different ball game. The stroke patient can also cook and knows what ingredients to use but a mentally ill person cannot do this [Young men, Gyegyeano]*

The second category believed that stroke is worse than other disabling conditions because of its multifaceted disabilities, and this formed the dominant theme. The narratives of the participants suggest that for someone with physical disabling condition, he/she may only have just that particular disability to deal with; however, stroke is usually associated with multiple disabilities which may be physical, mental, social, and cognitive. Another reason given was that people with physical disabling condition are better than those living with stroke because they are created by God and so they are not sick.

*There are differences. For the physically challenged person, he was born with and no one can really understand it. Someone could have gotten a leg problem from childhood. But for a stroke patient, he may not be able to do many things like bathing himself. For those with other physical disabling conditions, I don’t see them as people who are sick. They are the creation of God. [Elderly men, Tafo]*

The third category mentioned that post-stroke physical disability is better than other physical disabling conditions because a stroke sufferer can be restored back to his/her normal physical state with appropriate treatments. For instance, some of the participants said:

*There are differences. The physically challenged person who can’t walk may not be able to walk again but the one who has stroke can be cured and he can walk again. So there are differences. The stroke can be cured when you take medicines. You can’t compare that to someone who is physically challenged and may be maimed in the leg [Adult men, Tafo]*

Lastly, the adult women group from Tafo mentioned that there is no difference between stroke and other physical disabling conditions because both sufferers experience body breakdown or changes in body functions.

*Yes. They are the same. They are all handicapped or physically challenged so there is no difference between them [Adult women, Tafo]*

## Discussion

This study explored the cultural meanings of stroke in five communities in Ghana. All the words used to capture stroke in the five cultural settings centered on the physical disabilities associated with stroke after its onset, with no mention of other disabilities such as cognitive dysfunction, behavioural disability, psychological disability, etc. In addition, the dominant theme on what participants fear most about stroke was the post-stroke physical disability. This is similar to the study by Hundt *et al*., 2004 where the local words used by Shangaan Tsongan Mozambican refugees and South Africans in the Agincourt sub-district of Limpopo Province for the medical term ‘stroke centered on weakness in one side of the body or paralysis. The present findings suggest that the physical impact of stroke is more apparent and devastating in these communities. This is not surprising because even for stroke survivors, the physical impact of the illness constitutes the most important source of worry to them ( Sanuade, 2016). Stroke particularly brings about multifaceted disruptions which affect the life trajectories of the sufferers ( Bury, 1982; Becker, 1993; Kaufman, 1988; Owolabi & Ogunniyi, 2009; Sampane-Donkor *et al*., 2014). The implication of this is that interventions that address the physical impact of stroke may be easily welcomed in these communities. Also, this finding suggests the need to provide more education on the multifaceted impact of stroke in these communities.

Regarding stroke typology, participants had multiple ideas about stroke causation. Their narratives also suggest that adequate treatment of stroke depends on what the root cause of the illness is. While the ‘naturally caused’ strokes can be treated by physicians, ‘spiritually caused’ strokes can be treated either through faith or traditional healing. Furthermore, there was a general notion among the participants that stroke can be cured through early detection and treatment, use of herbal medicines, and availability of financial resources. The Ghana Health Service can capitalize on this by equipping local hospitals in such a way that early diagnosis could be made and patients could be stabilized before being referred to district or tertiary hospitals for further treatments. Previous research in Ghana showed that stroke has a huge financial impact on the sufferers and their families ( Sanuade, 2016); it is important that stroke treatment is covered in the National Health Insurance Scheme (NHIS) as this may improve the biomedical management of the condition. This can also be facilitated through the provision of NCD products such as laboratory tests and NCD care products at different hospitals (e.g. blood glucose meter, BP monitors) ( de-Graft Aikins *et al*., 2014). Regarding the use of herbal medicines as a treatment plan to cure stroke, the present findings reinforce the fact that the use of herbal medicine is part-and-parcel of the health seeking behaviour of Ghanaians ( Awuah *et al*., 2014; Okyerefo & Fiaveh, 2017), and will remain so for a long time. In the meantime, pharmacology tests to showcase ‘safe’ herbal medicines for stroke management needs to be a continued pursuit by the government.

The findings also suggest that participants’ perceptions on stroke causation and management are a mix of biomedical and socio-cultural/spiritual factors. However, primary intervention and management strategies in Ghana mostly focus on the biomedical explanations of illness and do not take the socio-cultural explanations into consideration. This partly explains why many of these interventions have been unsuccessful. An important way that may help to bridge the gap between the medical construction of stroke and the social representations of the illness in Ghana is making use of the community health workers, through the task-shifting framework. Since community health workers have been trained to provide care in dominant community health problems, they can equally be trained on stroke so as to increase their knowledge, confidence and ability to provide information, care, and support for local community residents ( de-Graft Aikins *et al*., 2014). This may consequently enhance primary prevention initiatives, acute care and rehabilitation and longer term social care for stroke in these communities.

Participants also believed that there are gradients in stroke severity. Generally, there was the notion that spiritual and left-sided strokes are more severe than natural and right-sided strokes, respectively. The findings suggest a high level of spiritual explanation of disease conceptualisations in these communities and this is because the activities of witchcraft, curse, and ghosts featured prominently in how stroke was generally perceived. Previous anthropological accounts in Ghana and other African countries showed that these activities are central to how an illness is perceived ( Ashforth, 2003; Evans-Pritchard, 1976; Field, 1937; Hundt *et al*., 2004; Moore & Sander, 2003). The findings also showed that these beliefs are still part of the social fabric of Ghanaians and reinforces Allan Young’s idea that there is a difference between biomedical diagnosis and sociological understandings and experience of an illness ( Young, 1976). Further, the findings underpin what these previous studies showed that illness causation and experience in an African setting is a mix of biological, psychological, social and cultural factors. It is important that these contexts are considered when developing intervention strategies on stroke in these communities. Based on medical perspective, stroke has two main types namely haemorrhagic and ischemic stroke, and their treatment strategies are radically different. None of the participants in this study differentiated between these two types of stroke; perhaps, they had no idea how they are different in terms of aetiology and management. It may be easier for local community residents to have more knowledge of this if the stroke types are classified as two separate diseases instead of one disease with two types. This is because previous engagements with some Ghanaian communities suggest that community residents think in terms of different disease conditions rather than different types of the same condition. They sometimes find it confusing to distinguish between types of a particular illness. Hence, in order to simplify identification of stroke types by ‘non-health professionals’, the possibility of classifying them as two separate conditions should be looked into by the medical community.

With respect to the groups at risk of stroke, the findings showed that there were age and gender dynamics. Regarding age, many had the notion that older people have higher risk of stroke, and this is consistent with what literature has shown ( Feigin *et al*., 2017; Zhang *et al*., 2012). Research showed that stroke incidence is higher among the elderly because they are more likely to have higher prevalence of classic vascular risk factors such as ischemic heart diseases, chronic heart failure, and atrial fibrillation, which build-up over time ( Yao *et al*., 2012). Concerning the gender dynamics on risk relativity, while the male groups mentioned that men are more at risk of stroke, the female groups stated that women have higher risk. This suggests that both gender perceived themselves as ‘victims of risk’. Research has shown that there are no inherent biological characteristics that predispose a particular gender to stroke; however, relativity of risk is dependent on different exposure to stroke risk factors, medical treatment, and therapeutic interventions ( Morović *et al*., 2012). Interventions that emphasise this information need to be promoted in these communities. An unexpected theme which emerged during the group discussions with young women was the mention of menstruation as a protective factor for stroke. This contradicts the findings from a study of 1,412 postmenopausal Japanese women ( Murakami *et al*., 2016). The study showed that during the 12 year follow-up (1998–2010), menstrual factor such as early menarche was associated with incidence of stroke and cerebral infarction ( Murakami *et al*., 2016). Since Murakami *et al*. (2016)’s findings cannot be extrapolated to Ghanaian setting, this study recommends the need for future studies to explore the association between menstrual factors and stroke, especially in the five communities.

Generally, participants mentioned that stroke is more severe than other disabling conditions due to the multifaceted disabilities associated with the illness. This corroborates what other research has shown, that stroke is a major cause of complex disability ( Adamson *et al*., 2004; Lai *et al*., 2003). Compared with other diseases, stroke is a major health problem that can cause multiple or concurrent disabilities in an individual ( Lai *et al*., 2003) which can influence all dimensions of life, including the simplest self-care tasks ( Adamson *et al*., 2004). In addition, participants perceived stroke to be more severe than other disabling conditions because while people with other mental or physical disabling conditions are not considered as sick because they are God’s creations, those with stroke are perceived as sick. This belief reinforces the constructionists’ idea that ‘there is nothing inherent about a condition that makes it stigmatizing; rather, it is the social response to the condition and some of its manifestations, or the type of individuals who suffer from it, that make a condition stigmatised’, This also supports the social model of disability that disability is determined by the way society is organised and cannot be reduced to a mere biological problem located in an individual’s body ( Barnes & Mercer, 2010).

## Study limitation

The major limitation of this study is that the findings may not be generalizable to all Ghanaian communities or ethnic groups. It is possible that people who belong to the same ethnic group but located in separate communities may perceive stroke differently. However, the multi-site approach adopted in this study revealed interesting variations in the social meanings of stroke across different geographical locations. These meanings can enhance development of context-specific interventions for stroke in other African countries.

## Conclusion

This study showed that the cultural meanings of stroke in five communities across Ghana are multifaceted and sometimes contradict the medical notion of the illness. Most often, people do not draw on one of either the cultural meanings or medical meaning, but can draw on these two meanings simultaneously or in a sequential manner. Since individuals and groups produce their own conceptions of reality and because knowledge itself is the product of social dynamics ( Conrad & Barker, 2010), it is important that intervention strategies, targeted at improving stroke prevention and control, need to pay critical attention to the cultural meanings of the illness. This is because culture is an important building block for how diseases are interpreted and managed ( Airhihenbuwa & Iwelunmor, 2012). Hence, if medicine as a practice or policies focus exclusively on the natural history of stroke, it may be difficult to ensure adequate primary prevention strategies and stroke rehabilitation in Ghanaian communities. Since there are usually ontological divides between patients and physicians, many of the people living with stroke live in cultural reality that is sometimes different from that of the physicians. Thus, it is important that stroke clinicians have a good understanding of the social and cultural landscapes of the communities in which they work so that they can, together with local communities, come up with context-specific interventions for stroke management and control.Therefore, studies which explore the cultural meanings of the different aspects of stroke (i.e. causes, prevention, and management) and the association between menstrual factors and stroke need more attention. This study also highlights the need to promote education on the multifaceted impact of stroke in Ghanaian communities. Finally, in addition to paying attention to the cultural meanings of stroke, other factors such as access to care, cost of health care, economic realities and constraints of poverty should be given much consideration in stroke prevention and control.

## Data availability

The full data for this study are not provided because the audio and transcripts of the interviews contain identifiable and sensitive information making it impossible to protect participants’ confidentiality. To apply for access to the data, please contact the author at o.sanuade@ucl.ac.uk. Data will only be shared if the following conditions are met. First, anyone who applies to access the data must maintain the confidentiality of the participants. Second, the identity of any individual or organization mentioned in or in relation to the data must not be disclosed. Third, the author of this article must be informed about any publication in which the data will be used. Finally, if the data is used in any other publication, it should be acknowledged that it is of secondary source, and appropriate citation must be provided.

## Supplementary material

Supplementary File 1: Data collection sheet for socio-demographic characteristics of participants.

Supplementary File 2: Focus group discussion (FGD) guide for local community residents.
