# Supplementary material for: Understanding the cultural meanings of stroke in the Ghanaian setting: A qualitative study exploring the perspectives of local community residents
Source: Wellcome Open Res. 2018 Nov 14;3:87. Originally published 2018 Jul 23. [Version 2] doi: 10.12688/wellcomeopenres.14674.2 (PMC6290971; doi:10.12688/wellcomeopenres.14674.2)
Supplement: Supplementary file 1 [file wellcomeopenres-3-16262-s0000.tgz › b51cdc41-8e8e-4e9c-9408-2fdaaa04ae96.docx]

| **Data collection sheet for socio-demographic characteristics of participants** | | | | | | | | | |
| --- | --- | --- | --- | --- | --- | --- | --- | --- | --- |
| **Name** | **Sex** | **Age** | **Religion** | **Marital Status** | **Education** | **Ethnicity** | **Occupation** | **Average monthly Income (GHC)** | **Contact number/Address** |
|  |  |  |  |  |  |  |  |  |  |
|  |  |  |  |  |  |  |  |  |  |
|  |  |  |  |  |  |  |  |  |  |
|  |  |  |  |  |  |  |  |  |  |
|  |  |  |  |  |  |  |  |  |  |
|  |  |  |  |  |  |  |  |  |  |
|  |  |  |  |  |  |  |  |  |  |
|  |  |  |  |  |  |  |  |  |  |
|  |  |  |  |  |  |  |  |  |  |
|  |  |  |  |  |  |  |  |  |  |
|  |  |  |  |  |  |  |  |  |  |
|  |  |  |  |  |  |  |  |  |  |
