# Supplementary material for: Understanding the cultural meanings of stroke in the Ghanaian setting: A qualitative study exploring the perspectives of local community residents
Source: Wellcome Open Res. 2018 Nov 14;3:87. Originally published 2018 Jul 23. [Version 2] doi: 10.12688/wellcomeopenres.14674.2 (PMC6290971; doi:10.12688/wellcomeopenres.14674.2)
Supplement: Supplementary file 2 [file wellcomeopenres-3-16262-s0001.tgz › 9658003b-cbec-46e5-97db-42918dfe5d32.pdf]

Focus Group Discussion (FGD) Guide for Local Community Residents

Introduction: – Identity and role of interviewer, general aims of research and role of participant in the process, issues of confidentiality, data access and ownership.

Socio-demographic details to be gathered on standardised form.

1. General Life History

Could you tell me a little bit about yourself?

Prompts: age, education, marital status, occupation, religious affiliation, etc.

Section A: Health and Illness

1. What does health means in your cultural context?
2. How do you think one can stay healthy in this community?
3. What does illness mean in your cultural context? *(Explore causes of illness).*
4. What are the common forms of illnesses in this community?
    a. Can you list the illnesses that can be cured and those which cannot be cured? *(Explore the causal theories of illnesses that can be cured and those which cannot be cured)*

Section B: Chronic Illness

1. How would you define/describe chronic illness?
2. How would you define noncommunicable disease?
    a. Is chronic disease different from noncommunicable diseases in your cultural context? *(Explore reasons)*
    b. What is/are the word(s) used to describe chronic/noncommunicable diseases in your dialect? (*Explore the origin of such word and why such word is used to describe chronic/noncommunicable diseases)*
3. What types of chronic illness do you know?

    Prompts**:** with names – hypertension, diabetes, stroke, asthma, arthritis, epilepsy, sickle cell, cancer, etc. *(Explore local words used for specific diseases)*

    (*When list is obtained, conduct sorting exercise to grade most severe to least severe illness, on a scale of 1 to 10; 1 being least severe and 10 being most severe*)

    Explore why illnesses have been graded the way they have been.
4. What do you think are the causes of chronic noncommunicable diseases*? (Explore if chronic noncommunicable diseases can be caused by infections or micro-organism and how this can happen)*

5. Do you live with any of these illnesses? *(Explore list)*
6. Can chronic noncommunicable illness be prevented? (*Explore how).*
    a. What are the things you have heard or know about how chronic noncommunicable diseases can be avoided? *(Explore sources of knowledge)*
    b. Are you able to adhere to this information? *[Explore the factors (individual/cultural/community) that enhance/impede adherence].*
7. Which group of people are most likely to get chronic noncommunicable illness and why?

   Prompts: Men/women; young/old, etc
8. Can chronic noncommunicable diseases be cured? *(Explore reasons).*
9. How can noncommunicable diseases be treated? (*Explore opinion of traditional vs biomedicine in treating chronic noncommunicable diseases or spiritual/psychological/physical forms of treatment)*
    a. How do people treat chronic noncommunicable diseases in this community?
10. What kinds of ideas or beliefs do people in this community have about chronic noncommunicable diseases?
    a. Does this affect the way you view people living with noncommunicable diseases?
11. Over the last 10 years, do you think there has been a change in the number of people living with chronic noncommunicable diseases in this community? *(Explore if there is an increase, decrease or constancy in chronic disease pattern)*
12. What do you think can be done to reduce the burden of chronic noncommunicable diseases in this community? *(Explore recommendations at the individual, family, community and systems' levels).*

Section C: Conceptualisations of Stroke

1. Have you heard of stroke before? *(Explore if they have ever suffered from stroke or have family members living with stroke or seen someone living with stroke).*
2. How will you define/describe stroke?
    a. What is/are the 'local' word(s) used to describe stroke in your dialect and what do these mean? *(Explore the origin of such word and why such word is used to describe stroke)*
    b. What are the symptoms of stroke?
3. Is stroke a chronic disease/noncommunicable disease? *(Explore reasons).*

4. Do you think stroke can be cured? *(Explore reasons).*
5. Generally, what are the causes of stroke?
    a. Do you think there is any relationship between stroke and other chronic and infectious diseases? (*Explore the nature of the relationship)*
    b. What are the illnesses that can cause stroke?
    c. What other illnesses can result from living with stroke?
6. How can one avoid getting stroke?
    a. What are the things you have heard or know about how stroke can be avoided? *(Explore sources of knowledge)*
    b. Are you able to adhere to this information? *[Explore the factors (individual/cultural/community) that enhance/impede adherence].*
7. Which group of people are most likely to get stroke? *(Explore reasons)*
   Prompts: *aged, men, women, young and old, etc.*
8. How can stroke be managed? (*Explore opinion of traditional vs biomedicine in treating stroke or spiritual/psychological/physical forms of treatment)*
    a. How is stroke being managed in this community?
9. What kinds of ideas or beliefs do people in this community have about stroke? *(Explore how these ideas constitute common sense knowledge and whether there has been a change in these ideas over time)*
    a. Does this affect the way you view people living with stroke?
10. What do you think are the impact of stroke? *(Explore the various disabilities that could result from stroke)*
    a. What are the chief problems of stroke?
    b. How severe is stroke? Will it have a short or long course?
    c. What do you fear most about stroke?
11. Do you think there is a difference between people living with stroke with different disabilities (physical, psychological, cognitive, etc) and people living with other forms of disabilities (physical, psychological, cognitive, etc) but are not living with stroke? *(Explore reasons)*

Section D: Social History of Chronic diseases

a) Alcohol

1. What do you think causes people to consume alcohol in this community?

2. What types of alcohol are mostly consumed in this community*? (Explore if types of drink consumed are based on social class)*
3. In what ways do you think alcohol can affect the health of an individual?
   a. Do you think there is an association between alcohol consumption and chronic disease*? (Explore nature of association)*
4. In what circumstances do people mostly consume alcohol in this community?

   Prompts: *during funerals, social gatherings, job loss and other economic disruptions, marital disruption, political campaigns, etc*
5. What roles does alcohol perform in this community? *(Explore views on religious functions, economic functions, political functions and social functions)*

b) Smoking

1. What do you think causes people to smoke in this community? *(Explore different types of smoking)*
2. In what ways can smoking affect the health of an individual?
   a. Do you think there is an association between smoking and chronic disease? *(Explore nature of association)*
3. In what circumstances do people mostly smoke in this community?

   Probes: *during funerals, social gatherings, job loss and other economic disruptions, marital disruption, etc*
6. What roles does smoking perform in this community? *(Explore views on religious functions, economic functions, political functions and social functions)*

c) Physical Activity

1. What does physical activity means to you? *(Explore if they have heard of the benefits of physical activity and find out the sources of knowledge)*
2. In what ways do you think an individual can be physically active?

   ***Prompts*:** through exercise, nature of jobs, etc.
3. How will you describe the level of physical activity in this community?
4. In your opinion, is there an association between physical activity and chronic diseases? *(Explore nature of association)*
5. What do you think hinders people in this community not to be physically active?

   ***Prompts*:** lack of physical activity space, nature of jobs, non-relevance to culture, etc.

d) Fruits and Vegetables

1. What types of fruits are available in this community? *(Explore list of fruits)*
    a. What fruits are available seasonally?
    b. What fruits are available throughout the year?
    c. Do you think fruits are easily accessible/affordable by everyone in this community?*(Explore reasons)*
2. What types of vegetables are being consumed in this community? *(Explore list of vegetables)*
    a. Do you think they are easily accessible/affordable by everyone in this community? *(Explore reasons)*
3. In what ways do you think fruits and vegetables affect the health of an individual?
    a. Is consumption of fruits and vegetables associated with chronic diseases? *(Explore reasons)*
    b. What are the other functions of fruits and vegetables?
    c. What types of health messages have you heard about benefits of fruits and vegetables? *(Explore sources of knowledge)*
    d. What hinders people in this community from adhering to such health information?
