# Supplementary material for: Understanding the cultural meanings of stroke in the Ghanaian setting: A qualitative study exploring the perspectives of local community residents
Source: Wellcome Open Res. 2018 Nov 14;3:87. Originally published 2018 Jul 23. [Version 2] doi: 10.12688/wellcomeopenres.14674.2 (PMC6290971; doi:10.12688/wellcomeopenres.14674.2)
Supplement: Peer review history

# Peer review: Referee response for version 2

**DOI:** 10.21956/wellcomeopenres.16262.r34259

**Published:** 2018-12-07

**Authors:** Christopher McKevitt

**Affiliations:** 1 School of Population Health & Environmental Sciences, King’s College London, London, UK

The author has addressed reviewer comments. The final paper is an important contribution to the literature.

I have read this submission. I believe that I have an appropriate level of expertise to confirm that it is of an acceptable scientific standard.

# Peer review: Referee response for version 2

**DOI:** 10.21956/wellcomeopenres.16262.r34260

**Published:** 2018-11-21

**Authors:** Gillian Lewando Hundt

**Affiliations:** 1 MRC/Wits Rural Public Health and Health Transitions Research Unit (Agincourt), School of Public Health, Faculty of Health Sciences, University of the Witwatersrand, Johannesburg, South Africa; 2 University of Warwick, Coventry, UK

I am happy with this revised version of this interesting paper which is a contribution to the field in this area.

I have read this submission. I believe that I have an appropriate level of expertise to confirm that it is of an acceptable scientific standard.

# Peer review: Referee response for version 1

**DOI:** 10.21956/wellcomeopenres.15976.r33721

**Published:** 2018-09-17

**Authors:** Christopher McKevitt

**Affiliations:** 1 School of Population Health & Environmental Sciences, King’s College London, London, UK

This paper is interesting and reports novel findings. It is relevant given the recognition of the significance of non-communicable disease in low and middle income countries. Apart from the historical overview presented in the background to the paper, there is relatively little research on how stroke is perceived from a social-cultural perspective. However, there is some research that could be referred to: for example, work from North America has illustrated how ‘culture’ – for example culturally informed ideas about aging - shapes the provision of stroke care and delineates the possibilities and perceived value of intervening ^1^^-^^4^.

 Consideration of this work might also help the author to review what appears to be a tendency in the paper to assume that while lay people may indeed draw on both cultural and scientific ideas to talk about stroke (page 12) stroke in scientific discourse is culture free.

 Some studies have reported research into cultural constructions of disability in Ghana and I wondered if these were relevant. I think it is unlikely that cultural understandings of stroke will exist entirely separately from wider constructions of disability, ageing, as well as disease and misfortune in general.

 The study engaged a large number of participants and a great deal of effort must have been made to recruit this large and diverse range of participants. It wasn’t entirely clear to me how participants were identified and invited to take part. Were any participants themselves stroke survivors or was this intended to collect information from the general population?

 I would have liked a bit more epidemiological and health service information about the relative significance of stroke, incidence, and current provision of care in Ghana.

 It might be useful to more clearly articulate implications for primary prevention initiatives, acute care and rehabilitation and longer term social care.

 Minor points

 What does it mean to say that ‘Medicine therefore appealed to the lay model in order to put the onus of recovery on the patient’ (page 3)?

 Page 8: ‘… in as much as such individual engages in poor lifestyles.’ – rephrase.

 One of the causes of stroke identified is thinking/worrying (page 8); does this equate to ‘stress’?

 Page 11: ‘Stroke particularly bring about…’:  ‘s’ missing

 Page 11: I wasn’t convinced by the suggestion that it would be easier for local community residents to have more knowledge of the different aetiologies of stroke if these were classified as two different types of stroke, rather than one disease with two types.

I have read this submission. I believe that I have an appropriate level of expertise to confirm that it is of an acceptable scientific standard, however I have significant reservations, as outlined above.

## References

[1] BeckerG: Continuity after a stroke: implications of life-course disruption in old age. *Gerontologist*.1993;33(2) :148-58 846800710.1093/geront/33.2.148

[2] KaufmanS: Illness, biography, and the interpretation of self following a stroke. *Journal of Aging Studies*.1988;2(3) : 10.1016/0890-4065(88)90002-3 217-227 10.1016/0890-4065(88)90002-3

[3] KaufmanSBeckerG: Content and Boundaries of Medicine in Long-Term Care: Physicians Talk About Stroke. *The Gerontologist*.1991;31(2) : 10.1093/geront/31.2.238 238-245 10.1093/geront/31.2.238 1904386

[4] HoffmannJ: *Urban Life and Culture*.1974;3(1) : 10.1177/089124167400300103 50-70 10.1177/089124167400300103

# Response

**Published:** 2018-11-06

**Authors:** Olutobi Sanuade

**Affiliations:** University College London, UK

Comment: Apart from the historical overview presented in the background to the paper, there is relatively little research on how stroke is perceived from a social-cultural perspective. However, there is some research that could be referred to: for example, work from North America has illustrated how ‘culture’ – for example culturally informed ideas about ageing - shapes the provision of stroke care and delineates the possibilities and perceived value of intervening ^1^ ^-^ ^4^. Consideration of this work might also help the author to review what appears to be a tendency in the paper to assume that while lay people may indeed draw on both cultural and scientific ideas to talk about stroke (page 12) stroke in scientific discourse is culture free.

**Response: I have cited the works of Hoffman (1974), Kaufman (1988), Kaufman and Becker (1991) and Becker (1993) in the article to draw out the fact that scientific discourse on stroke is not culture free.  I have also argued how culturally informed ideas shapes stroke management and the possibilities of intervening. This argument is made in the 2 ^nd^ paragraph under the introduction section.**

 Comment: Some studies have reported research into cultural constructions of disability in Ghana and I wondered if these were relevant. I think it is unlikely that cultural understandings of stroke will exist entirely separately from wider constructions of disability, ageing, as well as disease and misfortune in general.

**Response: In the Ghanaian literature, there has always been a distinction in how the society perceives people living with different forms of disability. While it is true that the constructions of stroke disability may not exist entirely separately from wider constructions of disability in Ghana, the focus of this study is to provide the ‘uniqueness’ of stroke disabilities from mental or physical disabilities. So I think the cultural understandings of stroke, even though falls within the broader conception of disability, is perceived differently from other conditions, just as the results showed.**

 Comment: The study engaged a large number of participants and a great deal of effort must have been made to recruit this large and diverse range of participants. It wasn’t entirely clear to me how participants were identified and invited to take part. Were any participants themselves stroke survivors or was this intended to collect information from the general population?

**Response: Participants were recruited through already established networks in these communities and were selected through a convenience method. Also, none of the participants was a stroke survivor and this was intended so as to gather information from the general population. This has been made clear in the paper.**

****

****

 Comment: I would have liked a bit more epidemiological and health service information about the relative significance of stroke, incidence, and current provision of care in Ghana.

**Response: I have added some information on the burden of stroke and the current provision of stroke care in Ghana in the methods section.**

 Comment: It might be useful to more clearly articulate implications for primary prevention initiatives, acute care and rehabilitation and longer-term social care.

**Response: A practical way of ensuring primary prevention intervention, acute care and rehabilitation and longer-term social care has been presented more clearly in the discussion and conclusion sections.**

 Comment: What does it mean to say that ‘Medicine therefore appealed to the lay model in order to put the onus of recovery on the patient’ (page 3)?

**Response: This sentence has been taken out because of the lack of clarity**

 Comment: Page 8: ‘… in as much as such individual engages in poor lifestyles.’ – rephrase.

**Response:** **This has been rephrased as:**

***There was a general consensus in all the group discussions that anyone can be at risk of stroke if such individual engages in poor lifestyles.***

 Comment: One of the causes of stroke identified is thinking/worrying (page 8); does this equate to ‘stress’?

**Response:** **This is different from stress because the women used the concept of stress differently from worrying/thinking**.

 Comment: Page 11: ‘Stroke particularly bring about…’:  ‘s’ missing

**Response:** **This has been addressed.**

 Comment: Page 11: I wasn’t convinced by the suggestion that it would be easier for local community residents to have more knowledge of the different aetiologies of stroke if these were classified as two different types of stroke, rather than one disease with two types.

**Response:** **I acknowledge the fact that this idea is debatable. However, previous engagements with some Ghanaian communities suggest that community residents think in terms of different disease conditions rather than different types of the same condition. They sometimes find it confusing to distinguish between types of a particular illness. Hence, in order to simplify identification of stroke types by ‘non-health professionals’, I suggest that the possibility of classifying them as two separate conditions should be looked into by the medical community.**

****

# Peer review: Referee response for version 1

**DOI:** 10.21956/wellcomeopenres.15976.r33729

**Published:** 2018-08-20

**Authors:** Gillian Lewando Hundt

**Affiliations:** 1 MRC/Wits Rural Public Health and Health Transitions Research Unit (Agincourt), School of Public Health, Faculty of Health Sciences, University of the Witwatersrand, Johannesburg, South Africa; 2 University of Warwick, Coventry, UK

This is an interesting qualitative study on the cultural meanings of stroke based on 30 focus groups in 5 areas of Ghana which used 13 interviewers and with the recorded interviews in five languages translated verbatim to English. The review of literature is adequate.

 I have a few comments - I will deal with the minor ones first and then the more substantive one.

 p.5 line 4 closely knitted: I think should be close knit.  It is clear that this group are Muslim in contrast to the other groups and this is not mentioned, neither is there any analysis that draws on this. Perhaps this is relevant?

 I would suggest that the article could be improved by reorganising the paper so that it follows the topic guide less in terms of order. The community views of stroke could be added to the first part of the results about thinking about stroke which then gives more of a coherent form to the paper.

 I think the results do not make the most of the data and occasionally are marred by a judgement that medical thinking is superior.

 This sentence demonstrates a judgement that this view is held because people did not know about stroke. This is at varience with the idea that social and cultural meanings of stroke are valid and important.....

 'In addition, the Ga Mashie participants referred to stroke as a ghost illness *“sisa hela”* - a term developed in the 1960s when community knowledge on causes and management of stroke was low.' 

 I would suggest you remove the sentence  which ends 'lay model in order to put the onus of recovery on the patient' as it makes no sense.

 It would be useful to distinguish between diagnosis of disease as part of the biomedical model and experience of illness as part of the sociological or anthropological understandings and experience of conditions. Alan Young's work is relevant here.

 There is clearly a difference between natural and spiritual strokes in terms of ideas of causation. This I think from a close reading is similar to other studies of plural ideas about illness and treatment. I think it should not to be conflated with the right and the left  or mild and severe. So I would suggest you move the natural and spiritual to the beginning of the section as this is about causation and then the characteristics follow. Also the treatment for the natural and spiritual may be different. This is a pluralistic way of thinking about stroke so that these ways of thinking and treating are often combined. There can be multiple ideas about causation - for the spiritual -The idea that it is a ghost illness is an important one as are the ideas about being cursed or of witchcraft. For the natural ideas for reasons that include diet, worrying, and hypertension are also important

 Views on Severity and treatment are something else.

 So I would suggest that the results are reordered with careful attention to the data and the story that is emerging.

 The ideas for intervention are unformed but could be developed further but perhaps not in this paper.

 These revisions will improve the paper which is interesting and should be published.

I have read this submission. I believe that I have an appropriate level of expertise to confirm that it is of an acceptable scientific standard, however I have significant reservations, as outlined above.

# Response

**Published:** 2018-11-06

**Authors:** Olutobi Sanuade

**Affiliations:** University College London, UK

Comment: p.5 line 4 closely knitted: I think should be close knit.  It is clear that this group are Muslim in contrast to the other groups and this is not mentioned, neither is there any analysis that draws on this. Perhaps this is relevant?

**Response: I have replaced closely-knitted with close-knit. The narratives of the participants were not influenced by religion and this was why the analysis did not draw on this. Nevertheless, I have added a statement at the methods section which indicates that Chansegu community residents were predominantly Muslims.**

Comment: I would suggest that the article could be improved by reorganising the paper so that it follows the topic guide less in terms of the order. The community views of stroke could be added to the first part of the results about thinking about stroke which then gives more of a coherent form to the paper.

**Response:** **The results have been reorganised. I have brought the community views on stroke earlier in the result section to provide a good context for socio-cultural views on stroke.**

Comment: ****I think the results do not make the most of the data and occasionally are marred by a judgement that medical thinking is superior. This sentence demonstrates a judgement that this view is held because people did not know about stroke. This is at variance with the idea that social and cultural meanings of stroke are valid and important.....

'In addition, the Ga Mashie participants referred to stroke as a ghost illness *“sisa hela”* - a term developed in the 1960s when community knowledge on causes and management of stroke was low.' 

**Response: I agree with this comment, although it was not my intention to show the superiority of medical thinking over that of the local communities. Sentences which demonstrate that m** **edical thinking is superior have been reworded. For instance, the above sentence was actually provided by the participants. The sentence has been reworded to show that this was an explanation given by the participants and not the author’s interpretation/judgement. The new sentence now reads as:**

***In addition, Ga Mashie participants referred to stroke as a ghost illness “*** ***sisahela*** ***”; they mentioned that this term was developed in the 1960s when community knowledge on causes and management of stroke was low.***

Comment: ****I would suggest you remove the sentence which ends 'lay model in order to put the onus of recovery on the patient' as it makes no sense.

**Response:** **This sentence has been taken out.**

Comment: It would be useful to distinguish between diagnosis of disease as part of the biomedical model and experience of illness as part of the sociological or anthropological understandings and experience of conditions. Alan Young's work is relevant here.

**Response**: **This comment has been addressed. A statement which brings out the difference between the biomedical diagnosis and sociological understandings and experience of illnesses has been included in the discussion section.**

Comment: There is clearly a difference between natural and spiritual strokes in terms of ideas of causation. This I think from a close reading is similar to other studies of plural ideas about illness and treatment. I think it should not to be conflated with the right and the left or mild and severe. So I would suggest you move the natural and spiritual to the beginning of the section as this is about causation and then the characteristics follow.

**Response: This comment has been addressed. In the results section, I have reordered the stroke types to reflect causation and characteristics.**

Comment: Views on severity and treatment are something else. So I would suggest that the results are reordered with careful attention to the data and the story that is emerging.

**Response:** **I have rearranged the results and discussion section to tell the stories that are emerging from the data.**
